# HOXA-AS2 may predict the prognosis of solid tumors among Chinese patients: A meta-analysis and bioinformatic analysis

**DOI:** 10.3389/fonc.2022.1030825

**Published:** 2022-10-31

**Authors:** Qiang Wang, Wei Zhang, Chao Deng, Shicheng Lin, Yejiang Zhou

**Affiliations:** ^1^ Department of Gastrointestinal Surgery, The Affiliated Hospital of Southwest Medical University, Luzhou, China; ^2^ Department of General Surgery, Jianyang People’s Hospital, Jianyang, China

**Keywords:** lncRNA, HOXA-AS2, cancer, prognosis, meta-analysis, bioinformatics analysis

## Abstract

**Background:**

HOXA cluster antisense RNA 2 (lncRNA *HOXA-AS2*) is a long noncoding RNA (lncRNA) that aberrantly expressed in various cancers and is closely associated with cancer progression. To overcome the limitation of small sample sizes that are inherent to single studies, a meta-analysis was conducted to explore the relationship between the expression level of *HOXA-AS2* and cancer prognosis.

**Methods:**

Correlational studies were retrieved by searching the databases of PubMed, Embase and Web of Science (up to August 10, 2022). The survival and prognosis data included overall survival (OS), and clinical parameters were gathered and analyzed.

**Results:**

Eighteen publications with 1181 patients who were diagnosed with solid tumors were ultimately included. The results showed that, compared with patients with low *HOXA-AS2* expression, patients with high *HOXA-AS2* expression tended to have poorer overall survival (OS) (HR= 2.52, 95% CI 1.87-3.38, P < 0.01) and shorter disease-free survival (DFS) (HR=7.19, 95% CI 3.20-16.17, P < 0.01). In addition, elevated *HOXA-AS2* expression indicated a larger tumor size (OR =2.43, 95% CI 1.53–3.88,P < 0.01), more advanced TNM stage (OR=3.85, 95% CI 2.79-5.31, P < 0.01), earlier lymph node metastasis (LNM) (OR = 4.41, 95% CI 3.05-6.39, P < 0.01) and distant metastasis (DM) (OR= 2.96, 95% CI 1.87-4.7, P < 0.01). Furthermore, *HOXA-AS2* expression was notassociated with age (OR=1.15, 95% CI 0.90-1.47), gender (OR=1.16, 95% CI 0.89-1.53), or tumor differentiation (OR=1.21, 95% CI 0.56-2.63). Moreover, aberrant *HOXA-AS2* expression was related to drug sensitivity in various types of cancers.

**Conclusion:**

The overexpression of *HOXA-AS2* predicted poor cancer prognosis in the Chinese population, including poor OS, DFS, TNM, LNM, and DM. *HOXA-AS2* could serve as a promising prognostic biomarker and therapeutic target.

**Systematic Review Registration:**

https://www.crd.york.ac.uk/prospero/, identifier CRD42022352604.

## 1 Highlights

1. Long noncoding RNA HOXA cluster antisense RNA 2 (***HOXA-AS2***) was revealed increasing expression in most of solid tumor tissues
2. Elevated ***HOXA-AS2*** expression predicts poor cancer prognosis.
3. ***HOXA-AS2*** could be served as potential therapeutic target and prognostic marker.
4. ***HOXA-AS2*** may take effect in chemotherapy and may be correlated with chemoresistance.s

## 2 Introduction

Cancer is one of the leading causes of death worldwide. Since its onset is hidden, early unobvious symptoms are not easy to detect; therefore, cancer has become a key issue of global concern and causes a heavy economic burden to the world every year (1, 2). The global cancer burden is predicted to reach 28.4 million cases by 2040 (an increase of 47% compared with 2020) according to cancer statistics (1). In recent decades, cancer treatment has evolved from exclusively surgical treatment to multidisciplinary treatment involving chemotherapy, radiotherapy, immunotherapy, and targeted therapy (3). However, the survival rate of cancer patients is still not optimal. Many researchers are beginning to explore new treatment options, and with the rapid advances in molecular medicine, cancer research has reached the level of genes and molecules (4, 5).

Over the past decade, many noncoding RNAs have been closely linked to cancer occurrence and development (6, 7). DNA transcription products include coding RNA and noncoding RNA; the latter occupies more than 90% of the RNA in cells (8). Due to the inability to encode proteins, noncoding RNA was once viewed as an “useless product” of DNA transcription. Nevertheless, there is growing evidence that noncoding RNA plays a role in many processes of cancer progression, including cell proliferation, invasion, autophagy, apoptosis, and epithelial-mesenchymal transition (9, 10).

*HOXA-AS2* is a 1048-bp long noncoding RNA located on human chromosome 7p15.2 between the HOXA3 and HOXA4 genes in the HOXA cluster (11). In recent years, an increasing number of publications have shown that *HOXA-AS2* is highly expressed in diverse malignancies, including oral squamous cell carcinoma (12, 13), nasopharyngeal carcinoma (14), thyroid cancer (15, 16), breast cancer (17, 18), lung cancer (19–22), gallbladder carcinoma (23), gastric cancer (24, 25), hepatocellular carcinoma (26, 27), pancreatic cancer (28), colorectal cancer (29), bladder cancer (30), prostate cancer (31), ovarian cancer (32), endometrial cancer (33), cervical cancer (34, 35), osteosarcoma (36, 37) and glioblastoma (38). Cell function tests showed that high *HOXA-AS2* expression can promote the proliferation and invasion of cancer cells, inhibit apoptosis, and affect the cancer cell cycle (21, 32). *HOXA-AS2* could function as a competitive endogenous RNA (ceRNA) affecting the distribution of microRNAs on their targets, which could contribute to tumor progression and occurrence (37, 39). Furthermore, an increasing amount of data have indicated a correlation between abnormal *HOXA-AS2* expression and clinicopathological features and prognosis, indicating its potential as a prognostic and therapeutic marker (22, 40, 41). However, owing to the small number of cases in a single study, the results have been inconclusive, as the prognosis reported in such studies do not represent all cancer patients equally. Therefore, this meta-analysis systematically evaluated the prognostic value of *HOXA-AS2* in cancer patients.

## 3 Materials and methods

### 3.1 Literature search

**Figure 1:**
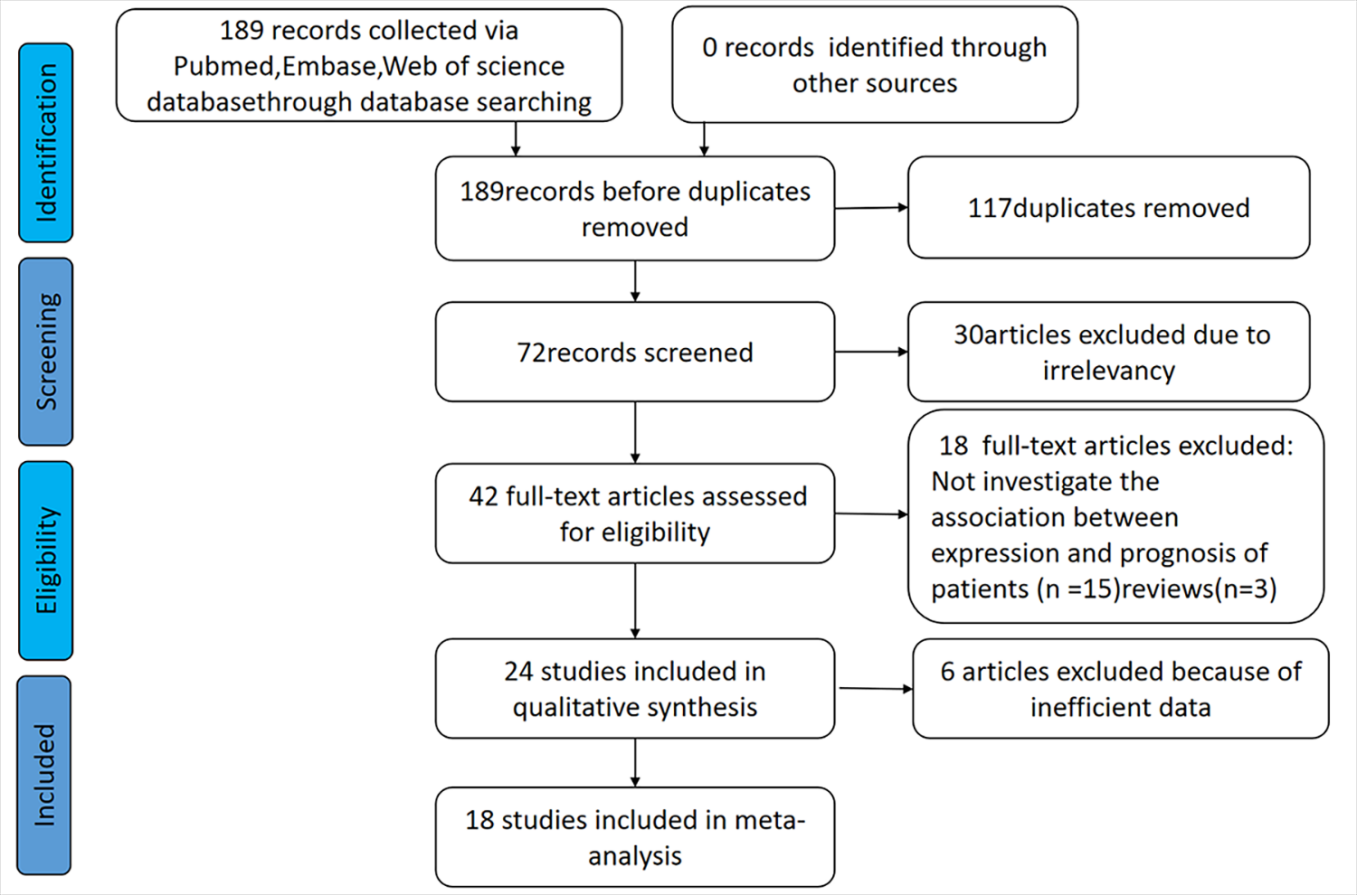
The flow diagram of the eligible studies.

This meta-analysis was conducted in accordance with the statement of the Preferred Reporting Items for Systematic Reviews and Meta-Analyses (42). The Embase, PubMed, and Web of Science databases were searched from inception to August 10, 2022. The search terms consisted of different subject terms, including: (“*HOXA-AS2*” or “Long noncoding RNA *HOXA-AS2*” or “LncRNA *HOXA-AS2*” or “HOXA cluster antisense RNA 2”) and (“neoplasm” or “cancer” or “carcinoma” or “tumor” or “malignancy”). The literature was limited to English-language publications and human studies (**Figure 1**). The study was registered on PROSPERO (registration number: CRD42022352604).

### 3.2 Inclusion and exclusion criteria of the literature

The inclusion criteria were as follows: 1) the research mainly focused on the relationship between the expression of *HOXA-AS2* and the clinicopathological characterization or prognosis of various solid human malignancies; 2) according to the expression of *HOXA-AS2*, patients were divided into two groups: a high expression group and a low expression group; 3) sufficient data were provided for the calculation of odds ratio (OR) or hazard ratio (HR) with 95% confidence interval (CI); and 4) the full text was published. The exclusion criteria were as follows: 1) nonprimary research articles, such as case reports, conferences, abstracts, reviews, or editorials; 2) patients were not divided into a high *HOXA-AS2* expression group and a low *HOXA-AS2* expression group; 3) data were not available or insufficient; and 4) animal experiments.

### 3.3 Quality evaluation

The Newcastle-Ottawa Scale (NOS) score (43) was applied to evaluate the quality of the included literature across three dimensions: Selection, Comparability and Outcome. The following aspects were evaluated: Adequate of case definition, Representativeness of the cases, Selection of Controls, Definition of Controls, Comparability of cases and controls, Ascertainment of exposure, Same method of ascertainment, Non-response rate. The total score ranges from 0~9, and studies with scores<6 are considered to be of substandard quality and were not included in this meta-analysis.

### 3.4 Data extraction and quality evaluation

In this study, two reviewers independently extracted the data (publication data) included in the article, and differences were resolved through discussion with a third reviewer. The data included the author’s name, publication year, country, cancer type, number of patients, sample type and detection method, high expression and number of patients with low expression, cut-off value, clinicopathological features, and follow-up times. The HR (95% CI) of survival was obtained directly from the study or calculated using Engauge Digitizer 11.1 software if Kaplan–Meier curves were available.

### 3.5 Statistical analysis

STATA 16.0 software was adopted for statistical analysis. We used the HR value and its 95% CI as the effect index of survival data (OS, DFS), and the odds ratio (OR) and its 95% CI as the clinical pathology of patients. When the Chi-squared test had a P < 0.10 or when I^2^ > 50%, the random effects model was selected, and the subgroup analysis was carried out according to the patient region, cancer type, data source method, etc. If P >0.10 or I^2^<50%, the fixed effects model was adopted.

### 3.6 Sensitivity analysis for publication bias

Publication bias of the literature was assessed using Egger’s test by applying STATA 16.0 software. The robustness of the results was tested by sensitivity analysis. P<0.05 was considered statistically significant.

### 3.7 Signal Pathway Network Construction, target gene and drug sensitivity prediction

Pathway Network Construction Related genes for *HOXA-AS2* were downloaded from the RNAInter v4.0 database (https://www.rnainter.org) (44). Next, Gene Ontology (GO) and Kyoto Encyclopedia of Genes and Genomes (KEGG) pathway enrichment analyses were conducted *via* the R package “clusterprofiler”. Cytoscape software (45) was used to construct a visualized signaling pathway network. Moreover, NCI-60 compound activity data and RNA-seq expression profiles from CallMiner™ were downloaded to analyze the drug sensitivity of HOXA-AS2 in pan-cancer (https://discover.nci.nih.gov/cellminer/home.do). Drugs approved by the FDA or clinical trials were adopted for analysis (46). The “ggplot2”, “impute”, and “ggpubr” R packages were used in R software.

## 4 Results

### 4.1 Characteristics of studies

The detailed process of the literature identification and selection is presented in **Figure 1**. The initial search resulted in 189 articles, of which 117 were excluded as they were duplicate studies. Another 30 articles were eliminated after screening the title and abstracts because of irrelevant findings. Further screening of the remaining 42 articles led to the exclusion of 18 reports due to a lack of study data regarding prognosis or clinicopathologic characteristics, and six were excluded due to missing data or information. Ultimately, 18 articles were included in the current meta-analysis.

A total of 18 articles including 1181 patients were included, and the cancer types included gastric cancer (24), colorectal cancer (CRC) (29, 40), hepatocellular carcinoma(HCC) (26, 27, 41), breast cancer (18), non-small cell lung cancer (NSCLC) (19, 22), prostate cancer (31), osteosarcoma (36, 37), bladder cancer (30), oral squamous cell carcinoma (OSCC) (13), papillary thyroid cancer (PTC) (15, 16), cervical cancer (35) and glioma (47). The publication years were from 2015 to 2021, and all patients were from China. The number of cases ranged from 27 to 128. Among the eighteen included studies, eleven studies provided overall survival (OS), 2 studies provided disease−free survival (DFS). Only 3 studies provided HR values directly, and other studies only provided K-M survival curves (**Table 1**). The NOS scores varied from 6-9 (**Supplementary Table S2**).

**Table 1:** Basic features of the publications included in this meta-analysis (n=18).

Study	Year	Region	Sample size	Cancer type	Detection method	Sample	Cut-off	survival analysis	HR statistics	Hazard ratios(95% CI)	Follow-up (month)	NOS score
Xie M	2015	China	55	Gastric cancer	qRT-PCR	tissue	median	OS	indirectly	6.45(1.92,21.63)	50	7
								DFS	indirectly	8.33(1.94,35.75)		
Li Q	2016	China	30	CRC	qRT-PCR	tissue	median	OS	indirectly	4.7(1.01, 21.87)	28	7
Ding J	2017	China	69	CRC	qRT-PCR	tissue	not reported	lack off	NA	-	NA	6
Wang F	2016	China	112	HCC	qRT-PCR	tissue	median	OS	indirectly	1.96(1.17,3.28)	80	7
Zhang Y	2018	China	58	HCC	qRT-PCR	tissue	mean	lack off	NA	–	NA	6
Lu Q	2020	China	116	HCC	qRT-PCR	tissue	median	OS	directly	2.76(1.115,6.848)	25	9
Wang Y	2018	China	66	Osteosar- coma	qRT-PCR	tissue	median	lack off	NA	–	NA	6
Wang L	2019	China	27	Osteosar- coma	qRT-PCR	tissue	mean	OS	indirectly	0.75(0.16,3.45)	60	7
Wu L	2019	China	50	Glioma	qRT-PCR	tissue	not reported	lack off	NA	–	NA	6
Fang Y	2017	China	38	Breast cancer	qRT-PCR	tissue	not reported	OS	indirectly	1.51(0.53, 4.27)	90	7
Li Y	2017	China	103	NSCLC	qRT-PCR	tissue	median	OS	directly	6.711(3.526-17.019)	75	9
								DFS	directly	6.737(2.926-20.527)		
Cui T J	2019	China	40	NSCLC	qRT-PCR	tissue	not reported	OS	indirectly	1.70(0.29,10.48)	75	8
Xia F	2018	China	128	PTC	qRT-PCR	tissue	mean	lack off	NA	–	NA	6
Jiang L	2019	China	68	PTC	qRT-PCR	tissue	mean	OS	indirectly	1.45(0.53, 3.98)	60	7
Wang F	2019	China	80	Bladder cancer	qRT-PCR	tissue	not reported	lack off	NA	–	NA	6
Xiao S	2020	China	68	Prostate cancer	qRT-PCR	tissue	mean	OS	indirectly	1.55(0.2, 11.91)	60	7
ChenR (a)	2021	China	46	OSCC	qRT-PCR	tissue	not reported	lack off	NA	–	NA	6
ChenR (b)	2021	China	27	Cervical cancer	qRT-PCR	tissue	mean	OS	directly	2.80(1.0, 7.9)	120	8

CRC, colorectal cancer; HCC, hepatocellular carcinoma; NSCLC, non-small cell lung cancer; PTC, papillary thyroid cancer; OSCC, Oral Squamous Cell Carcinoma; NA, not available; qRT-PCR, quantitative reverse transcription-polymerase chain reaction; OS, overall survival; DFS, disease−free survival.

### 4.2 Association between *HOXA-AS2* and prognostic indicators

#### 4.2.1 Association between HOXA-AS2 expression and OS

**Figure 2:**
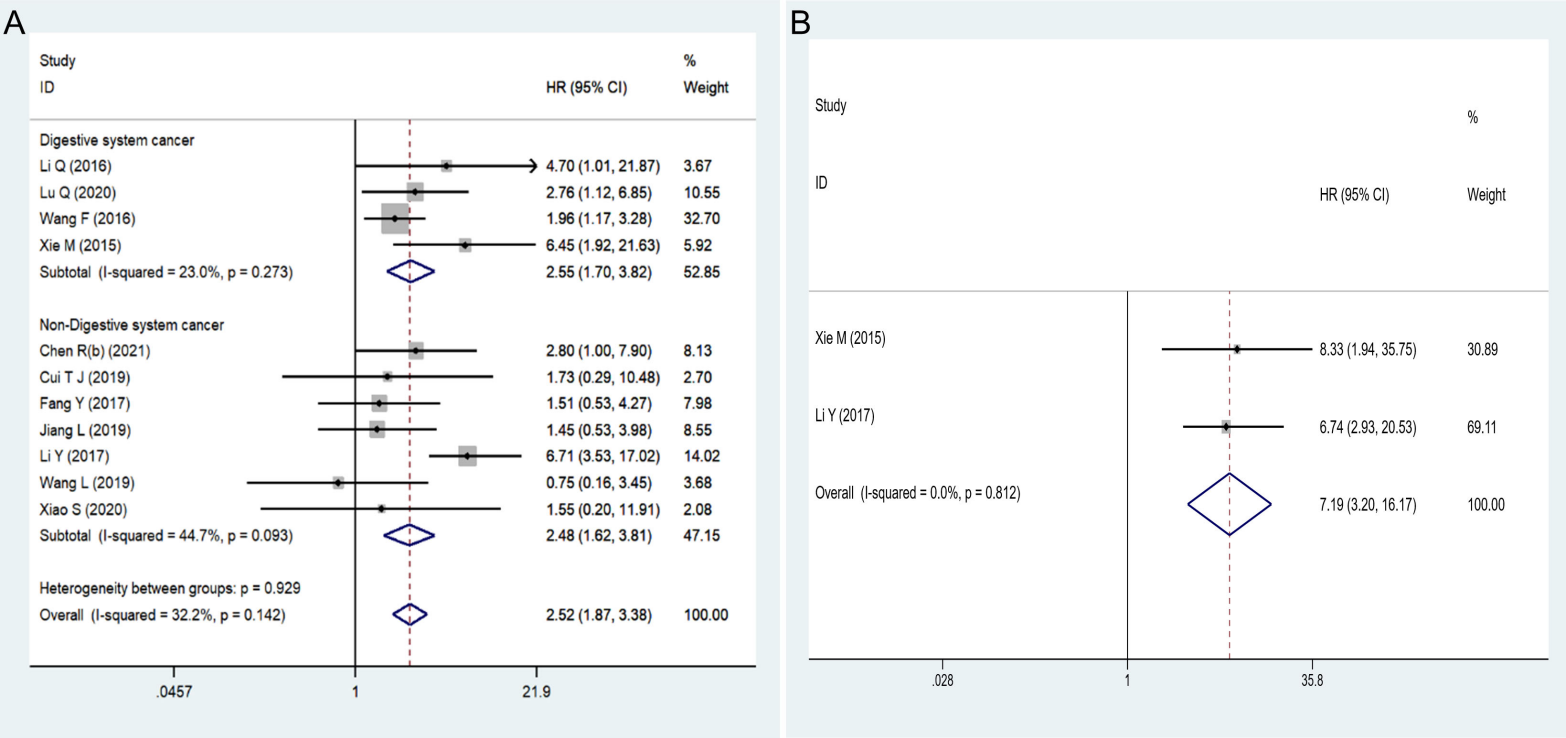
Forest plots showed: **(A)** the correlation between *HOXA-AS2* expression and overall survival (OS). **(B)** the correlation between *HOXA-AS2* expression and disease-free survival (DFS).

Eleven articles involving 684 patients reported the correlation between the expression level of *HOXA-AS2* and the prognosis of cancer patients. No noticeable heterogeneity was observed among the studies (I^2 =^ 32.2%, P=0.142), and therefore, fixed-effects model was utilized to estimate the HR of the studies. The results showed that high *HOXA-AS2* expression predicts poor cancer survival (pooled HR=2.52, 95% CI 1.87-3.38, P < 0.01). Subgroup analysis was adopted based on the tumor type, HR estimation method, sample size, follow-up time and tumor tissue origins (mesenchymal tissue, epithelial tissue), which further confirmed that *HOXA-AS2* overexpression was associated with poorer OS among cancer patients in all subgroups except for mesenchymal tissue origins (osteosarcoma) (HR=0.75, 95% CI 0.16-3.48) (**Figure 2A** **,** **Table 2**).

**Table 2:** Subgroup analysis of the pooled HRs with HOXA-AS2 expression in patients with malignancy.

		No. of studies	No. of patients	Pooled HR (95% CI)		Heterogeneity	
				Fixed	Random	*I^2^(%)*	*P-value*
**Overall survival(OS)**		11	684	2.52 (1.87-3.38)	2.54 (1.72-3.76)	32.2	0.142
**Cancer type**							
Digestive system		4	313	2.55 (1.70-3.82)	2.79 (1.67-4.68)	23	0.273
Non-digestive system		7	371	2.48 (1.62-3.81)	2.17 (1.18-3.99)	44.7	0.093
**HR estimation method**							
Indirectly		8	438	2.00 (1.39-2.86)	2.00 (1.38-2.88)	1.5	0.418
Directly		3	246	4.06(2.42-6.79)	3.96 (2.15-7.28)	27.3	0.253
**Sample size**							
≥50		6	522	2.75 (1.95-3.87)	2.94 (1.69-5.09)	51.7	0.066
<50		5	162	1.97 (1.11-3.51)	1.97 (1.11-3.51)	0	0.484
**Follow-up time(month)**							
≥60		8	483	2.25 (1.62-3.13)	2.17 (1.36-3.47)	38.2	0.125
<60		3	201	3.91 (2.03-7.53)	3.91 (2.03-7.53)	0	0.529
**Tumor Tissue origins**							
Mesenchymal tissue		1	27	0.75 (0.16-3.48)	0.75 (0.16-3.48)	–	–
Epithelial tissue		10	657	2.64 (1.95-3.56)	2.72 (1.86-3.96)	26.6	0.199
**DiseaseFree Survival (DFS)**		2	158	7.19 (3.20-16.17)	7.19 (3.20-16.17)	0	0.812

#### 4.2.2 Association of *HOXA-AS2* expression with DFS

Only two studies reported DFS data that could be used to assess the prognostic value of *HOXA-AS2.* Our analysis suggested that *HOXA-AS2* overexpression was associated with DFS (pooled HR = 7.19, 95% CI 3.20-16.17, P < 0.01) (**Figure 2B** **,** **Table 2**). No obvious heterogeneity was observed among the studies (I^2^ = 0%, P = 0.812).

### 4.3 Association between *HOXA-AS2* and clinicopathological features

#### 4.3.1 Association between *HOXA-AS2* expression and age, gender

**Figure 3:**
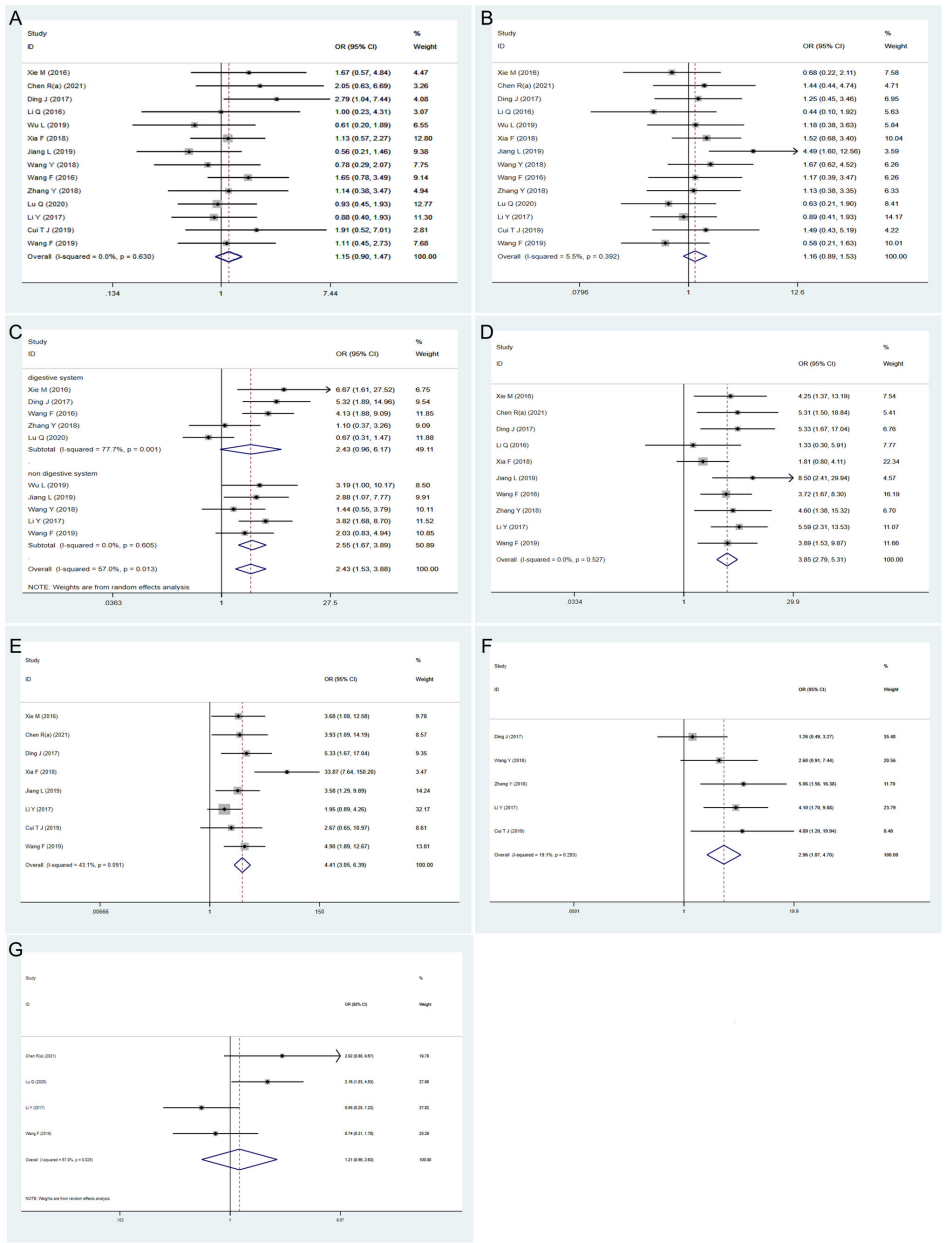
Forest plots showing the correlation between *HOXA-AS2* expression and clinicopathological parameters. **(A)** age. **(B)** gender. **(C)** tumor size. **(D)** TNM stage. **(E)** lymph node metastasis. **(F)** distant metastasis. **(G)** differentiation.

Fourteen studies with 1021 patients were examined to determine the association between the expression level of *HOXA-AS2* and age (**Figure 3A**). No obvious heterogeneity was observed (I^2^ = 0%, P=0.630); therefore, the fix-effects model was conducted. We found that there was no significant association between *HOXA-AS2* expression and patient age (OR=1.15, 95% CI 0.90-1.47, P > 0.05). Fourteen studies with 1021 patients investigated potential associations between *HOXA-AS2* expression and gender. Upon assessment with the fixed model, no obvious heterogeneity was observed amongst the studies (I^2^ = 5.5%, P=0.392). The expression of *HOXA-AS2* was not correlated with patient gender (OR=1.16, 95% CI 0.89-1.53, P > 0.05) **(** **Figure 3B** **).**

#### 4.3.2 Association of *HOXA-AS2* expression with tumor size

The relationship between tumor size and *HOXA-AS2* expression was evaluated in 10 studies involving 777 patients. There was obvious heterogeneity (I^2 =^ 57%, P=0.013); therefore, the random-effects model was carried out, and subgroup analysis was performed. The combined results indicated a significant positive correlation between high *HOXA-AS2* expression and larger tumors (OR=2.43, 95% CI 1.53-3.88, P < 0.01). Subgroup analysis revealed, a strong association between *HOXA-AS2* expression and tumor size in non-digestive system malignant tumor (OR =2.55, 95% CI 1.67-7.74, P < 0.01). However, no significant correlation was observed between *HOXA-AS2* expression and digestive system malignancies (OR=2.43, 95% CI 0.96–6.17, P > 0.05), indicating that the digestive system tumors may be the source of the heterogeneity (**Figure 3C**).

#### 4.3.3 The relationship between *HOXA-AS2* expression and TNM and lymph node metastasis (LNM)

There were 10 and 8 studies reporting the TNM stage and LNM in patients with solid tumors, respectively. The fix-effects model was carried out to show that the expression level of *HOXA-AS2* was significantly correlated to TNM stage (OR=3.85, 95% CI 2.79-5.31, P < 0.01) and LNM (OR = 4.41, 95% CI 3.05-6.39, P < 0.01) with no obvious heterogeneity (**Figures 3D, E**).

#### 4.3.4 The relationship between the expression of *HOXA-AS2* and distant metastasis (DM), tumor differentiation

A total of 5 qualified studies examined the occurrence of DM in patients with solid tumors. Since there was no significant heterogeneity, we used the fixed-effects model (I^2^ = 19.1%, P = 0.293) to analyze the DM data. The results suggested that the increased expression of *HOXA-AS2* was significantly related to DM (OR=2.96, 95% CI 1.87-4.7, P < 0.01) (**Figure 3F**). Four of the included studies reported the differentiation of tumors, and the random-effects model was used to analyze the relationship between *HOXA-AS2* expression and tumor differentiation (I^2 =^ 67.9%, P=0.025). We found that the increased expression of *HOXA-AS2* was not significantly correlated with the tumor differentiation (OR=1.21, 95% CI 0.56-2.63, P > 0.05) (**Figure 3G**).

### 4.4 Sensitivity analysis and publication bias

**Figure 4:**
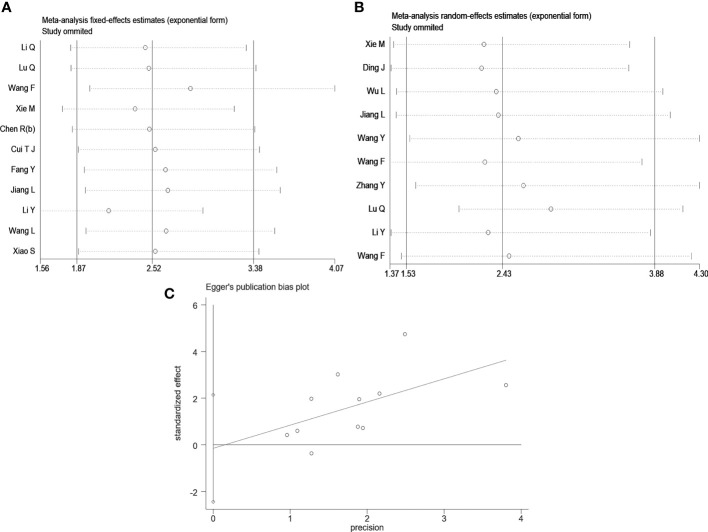
Sensitivity analysis and Egger’s test. **(A)** Sensitivity analysis for OS; **(B)** Sensitivity analysis for tumor size; **(C)** The Egger’s test and linear regression plot for the publication bias for OS.

Sensitivity analysis was conducted by the leave-one-out method to assess the effect of an individual article on the pooled OS and tumor size data. However, no single article significantly altered the results (**Figures 4A, B**). Egger’s test did not reveal evident publication bias for OS (Pr>|t|=0.884) **(** **Figure 4C** **)**, age (Pr>|t|=0.694), gender (Pr>|t|=0.524), tumor size (Pr>|t|=0.399), TNM stage (Pr>|t|=0.582), LNM (Pr>|t|=0.088), DM (Pr >|t|=0.507), or differentiation (Pr>|t| =0.697) (**Supplementary Figure S1**).

### 4.5 Prediction of *HOXA-AS2* function and drug sensitivity in pan-cancer

**Figure 5:**
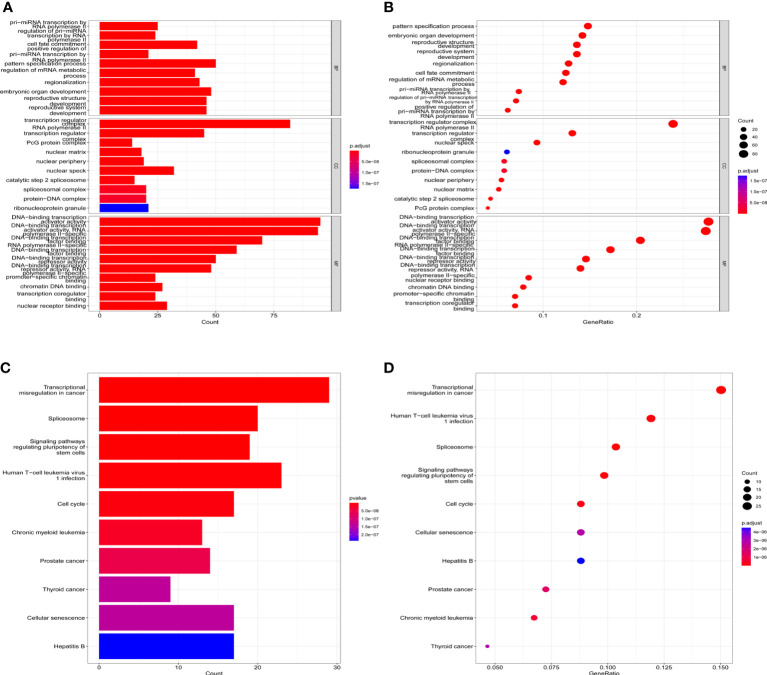
KEGG and GO term enrichment for HOXA-AS2. **(A)** Barplots of GO enrichment. **(B)** Bubble charts of GO enrichment; **(C)** Barplots of KEGG molecular mechanisms. **(D)** Bubble charts of KEGG molecular mechanisms.

**Figure 6:**
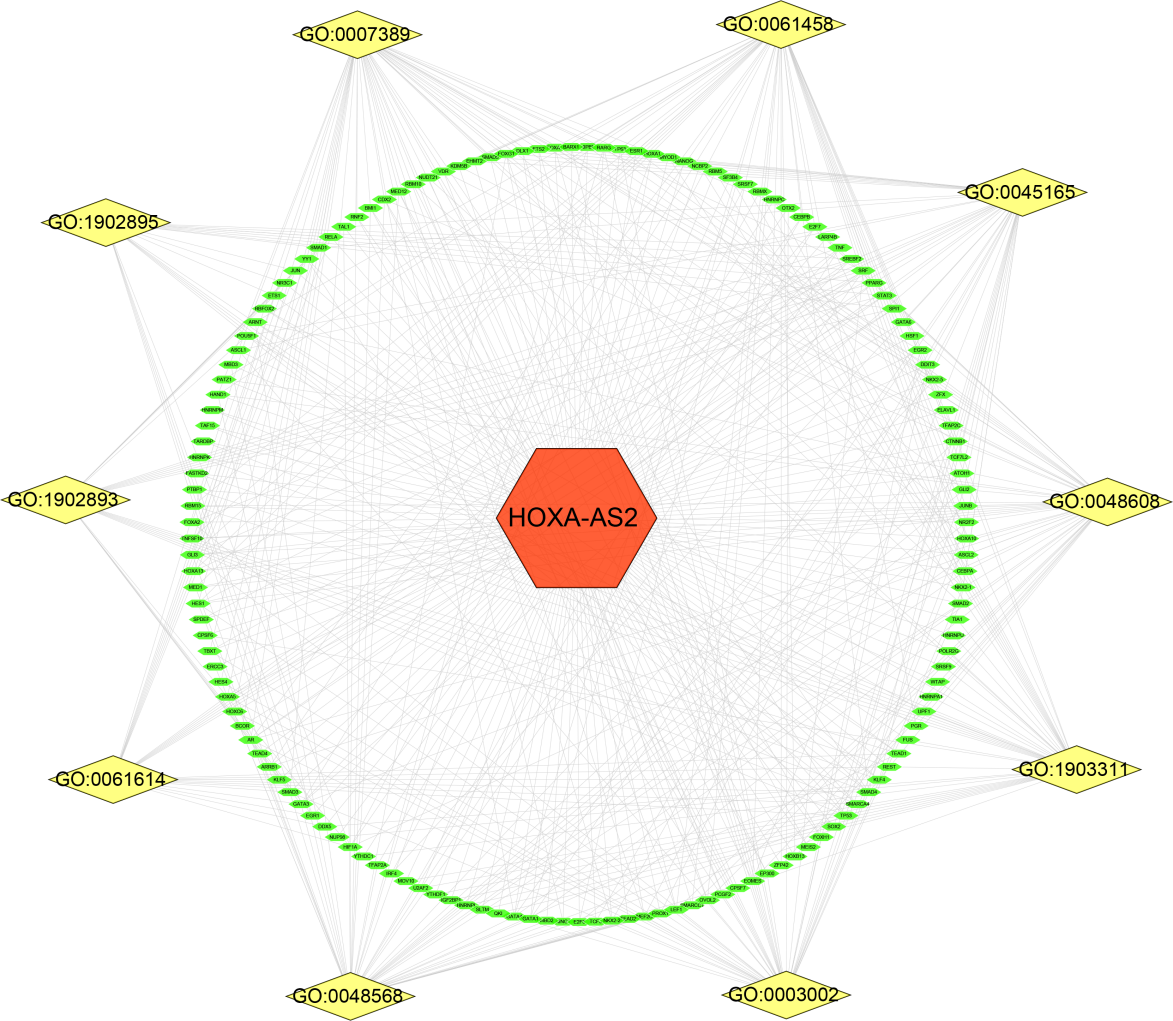
Related gene interaction network analysis. Yellow nodes represent the related pathway and green nodes represent target genes. GO:0061614 (pri-miRNA transcription by RNA polymerase II); GO:1902893 (regulation of pri-miRNA transcription by RNA polymerase II); GO:0045165 (cell fate commitment); GO:1902895 (positive regulation of pri-miRNA transcription by RNA polymerase II); GO:0007389 (pattern specification process); GO:1903311 (regulation of mRNA metabolic process); GO:0003002 (regionalization); GO:0048568 (embryonic organ development); GO:0048608 (reproductive structure development); GO:0061458 (reproductive system development).

Public databases were used to predict the biological functions and molecular mechanisms of *HOXA-AS2*. First, we used the RNAInter v4.0 database to screen interacting genes of *HOXA-AS2*(Confidence Score screening: 0.2-1.0), and 344 protein-coding genes were screened (**Supplementary Table S2**). **Figure 5** shows the underlying molecular mechanisms identified by GO and KEGG pathway analyses. *HOXA-AS2* was predicted to be mainly involved in biological processes (BPs), such as pri−miRNA transcription by RNA polymerase II, cell fate commitment, and embryonic organ development; molecular functions (MFs), such as DNA−binding transcription activator activity, RNA polymerase II-specific DNA-binding transcription factor binding; and cellular components (CCs), such as the transcription regulator complex, RNA polymerase II transcription regulator complex (**Figures 5A, B**). Additionally, the KEGG pathway analysis results showed that interacting genes were principally involved in transcriptional misregulation in cancer, the spliceosome, and signaling pathways regulating the pluripotency of stem cells (**Figures 5C, D**). Furthermore, Cytoscape software was applied to construct the top 10 visual signaling pathway networks (**Figure 6** **)**.

**Figure 7:**
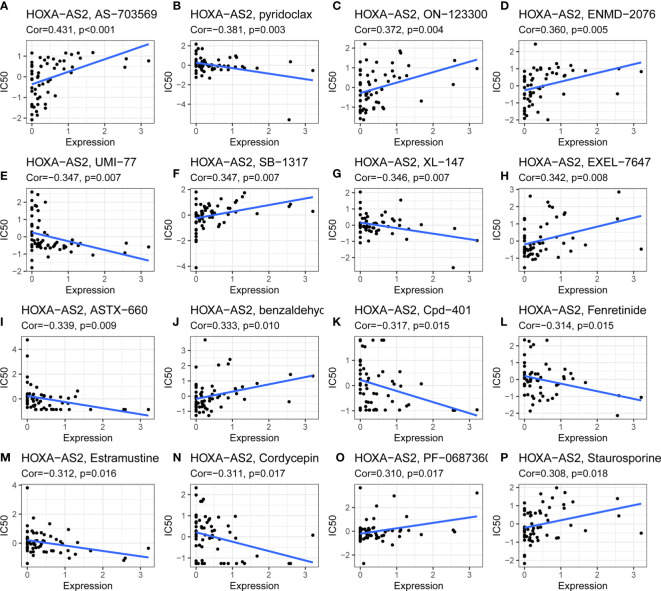
Drug sensitivity analysis of HOXA-AS2. The expression of HOXA-AS2 was associated with the sensitivity of **(A)** AS-703659, **(B)** pyridoclax, **(C)** ON-123300, **(D)** ENMD-2076, **(E)** UMI-77, **(F)** SB-1317, **(G)** XL-147, **(H)** EXEL-7647, **(I)** ASTX-660, **(J)** benzaldehyc, **(K)** Cpd-401, **(L)** Fenretinide, **(M)** Estramustine, **(N)** Cordycepin, **(O)** PF-0687360, **(P)** Staurosporine.

Using CellMiner™, we further investigated the potential correlation between *HOXA-AS2* expression and drug sensitivity. Notably, *HOXA-AS2* expression was positively correlated with the drug sensitivity of AS-703659, ON-123300, ENMD-2076, SB-1317, EXEL-7647, benzaldehyc, PF-0687360, and staurosporine (**Figures 7A, C, D, F, H, J, O, P**). Our results showed that *HOXA-AS2* expression was negatively associated with pyridoclax, UMI-77, XL-147, ASTX-660, Cpd-401, fenretinide, estramustine, and cordycepin (**Figures 7B, E, G, I, K–N**). The data suggested that *HOXA-AS2* might contribute to the chemoresistance development. Such as AS-703659 and Staurosporine. Most of the above positively correlated drugs were enzyme inhibitors, indicating that the involvement of *HOXA-AS2* in chemoresistance might be related to the metabolism of enzymes and transcriptional misregulation.

## 5 Discussion

In recent years, lncRNAs have attracted increasing attention due to rapid advances in high-throughput sequencing technology (48, 49). Increasing evidence suggests that abnormal lncRNA expression is related to human cancers, and dysregulation of lncRNAs contributes to tumor development by promoting cell proliferation, invasion and metastasis of tumor cells (50, 51). Some studies have reported that lncRNAs,such as LUCAT1 (52), SNHG7 (53), TP73-AS1 (54), are promising to be the potential therapeutic targets and biomarkers for diagnosis, prognostic evaluation or treatment. The prognostic value of *HOXA-AS2* expression levels for cancers are unclear, and the underlying mechanism has yet to be elucidated.

*HOXA-AS2* was found to be highly expressed in promyelocytic leukemia for the first time (55) and has been confirmed to be dysregulated in diverse tumor cells, such as osteosarcoma (37), glioma (56), NSCLC (20), CRC (57), and cervical cancer (35). It has also been proven to be involved in the biological behavior of many tumor cells as a high-risk factor. In osteosarcoma and cervical cancer, cell function tests show that *HOXA-AS2* upregulation promotes tumor cell metastasis, prevents the cell cycle, and epithelial-mesenchymal transition and inhibits apoptosis. Silencing *HOXA-AS2* can inhibit the above biological processes (35, 37). In hepatocellular carcinoma, *HOXA-AS2* expression was significantly increased in both cell lines and HCC tissues and was associated with poor survival and prognosis (41). Therefore, most studies reported that high expression of *HOXA-AS2* carried a higher risk of cancer progression and a poorer survival benefit.

Due to the existing research foundations and the urgent need to improve cancer prognosis, this study evaluated the prognostic value of *HOXA-AS2* for various solid tumors. A total of eighteen studies involving 1181 patients and 12 solid tumors were included. The results showed that high expression of *HOXA-AS2* was positively correlated with the poor prognosis of patients, including overall survival (HR=2.52, 95% CI 1.87-3.38, P<0.01) and disease-free survival (HR=7.19, 95% CI 3.20-16.17, P<0.01). Given the differential expression of *HOXA-AS2* in different tumor tissues, a subgroup analysis for OS was performed based on different tissue sources such as cancer types, different data extraction methods, sample sizes, follow-up times and tumor tissue origins, and there was no noticeable heterogeneity. However, we found no correlation between *HOXA-AS2* expression and mesenchymal tissue origins (osteosarcoma) (HR=0.75, 95%CI 0.16-3.48), which may be because only one small-sample study (n=27) was conducted in this subgroup or because of tumor heterogeneity between mesenchymal tumors and epithelial tumors. Therefore, further studies with a larger sample size are needed to validate the prognostic significance of *HOXA-AS2* in mesenchymal tumors.

Moreover, we analyzed the relationship between *HOXA-AS2* expression levels and clinical attributes. The results of the combined OR showed that elevated *HOXA-AS2* expression had no significant correlations with age, sex or tumor differentiation but was positively correlated with advanced TNM stage, earlier lymph node metastasis and distant metastasis. Overall, our data suggested that *HOXA-AS2* could serve as a potential reliable tumor prognostic indicator.

Previous studies have demonstrated that *HOXA-AS2* is highly expressed in various cancer tissues and closely associated with the prognosis of tumor patients, such as gastric cancer, hepatocellular carcinoma, non-small cell lung cancer, and cervical cancer. Some researchers have suggested that long noncoding RNAs could function as therapeutic targets for cancer and as independent indicators of tumor progression. Liu Y et al. reported that *HOXA-AS2* could promote cell proliferation, migration and invasion and inhibit apoptosis in NSCLC *via* the miR-520a-3p/HOXD8/MAP3K2 axis (58). Zheng et al. revealed that lnc*HOXA-AS2* expression was increased in NSCLC tissues and that *HOXA-AS2* could enhance the migratory and invasive abilities of tumor cells by upregulating IGF2 (20). Wang F et al. revealed that *HOXA-AS2* knockdown suppresses the migration, invasion and stemness of bladder cancer cells by regulating miR-125b/Smad2E signaling axis (30). In colorectal cancer, Ding et al. found that silencing *HOXA-AS2* could promote apoptosis, inhibit the proliferation and migration by binding to EZH2/LSD1, and upregulated *HOXA-AS2* is positively correlated with larger tumor size, advanced tumor stage and early lymph node metastasis. Wu Q et al. reported that *HOXA-AS2* may promote the cell proliferation and migration of cervical cancer cells by modulating the Notch pathway (34). Gao Y et al. identified that *HOXA-AS2* can play a role in glioblastoma cell viability, invasion, migration, and vasculogenic mimicry by interacting with EFGR *via* sponging miR-373 (59). Additionally, we explored the mechanisms of *HOXA-AS2* expression in ovarian cancer, gallbladder carcinoma, pancreatic cancer, and nasopharyngeal carcinoma and found similar results. These studies demonstrated that *HOXA-AS2* is capable of oncogenic activity. It has been shown that *HOXA-AS2* is related to various functions, including post-transcriptional regulation (56) and regulation of the pluripotency of stem cells (30). An overview of the molecular mechanisms governing *HOX-AS2*’s effects on various solid malignancies can be found in **Table 3**.

**Table 3:** Regulation mechanism of HOXA-AS2 involved in various solid tumors.

Cancer type	Expression	Micro-RNAs	Targets	Functions	References
NSCLC	up-regulation	miR-216a-5p	–	Proliferation, migration, invasion	Cui T J (19)
	up-regulation	miR-520a-3p	HOXD8/MAP3K2	cell proliferation, migration, invasion	Liu Y (58)
	up-regulation	miR-302a-3p	–	invasion, migration, proliferation	Pan X (21)
	up-regulation	-	IGF2	invasion, migration	Zheng (20)
Bladder cancer	up-regulation	miR-125b	Smad2	migration, invasion, stemness	Wang F (30)
Prostate cancer	up-regulation	miR-5590-3p	PBX3	EMT, proliferation, migration	Xiao S (31)
CRC	up-regulation	–	EZH2, LSD1	cell proliferation, inhibit apoptosis	Ding J (29)
HCC	up-regulation	miR-520c-3p	GPC3	cell proliferation, migration, invasion	Zhang Y (27)
hepatoblastoma	up-regulation	miR-217	HOXA3	proliferation, migration, invasion	Liu G (60)
Ovarian cancer	up-regulation	miR-520a-3p	–	proliferation,migration, invasion	Xie D (32)
Endometrial cancer	up-regulation	miR-302c-3p	ZFX,YKL-40	proliferation, invasion	Song N (33)
Breast cancer	up-regulation	miR-520c-3p	TGFBR2, RELA	proliferation, invasion	Fang Y (18)
Cervical cancer	up-regulation	miR-509-3p	BTN3A1	invasion, metastasis	Chen R (35)
	up-regulation	–	Notch Pathway	cell proliferation, migration	Wu Q (34)
Gastric cancer	up-regulation	–	P21/PLK3/DDIT3	cell proliferation, tumorigenesis	Xie M (24)
Osteosarcoma	up-regulation	miR-520c-3p	–	EMT, migration, invasion	Wang Y (37)
	up-regulation	miR-124-3p	E2F3	migration, invasion	Wang L (36)
Gallbladder carcinoma	up-regulation	–	E-cadherin	migration, invasion, EMT	Zhang P (23)
Glioma	up-regulation	miR-373	EFGR	migration, invasion, vasculogenic mimicry	Gao Y (59)
	up-regulation	–	RND3	proliferation, invasion, inhibit apoptosis	Wu L (47)
	up-regulation	miR-184	COL6A2	cell proliferation	Chen P Y (61)
	up-regulation	–	E2F8,E2F1,ATF3	proliferation,inflammation	Le Boiteux (56)
	up-regulation	miR-302a	KDM2A/JAG1	T cell proliferation, immune tolerance	Zhong C (62)
Glioblastoma	up-regulation	miR-885-5p	RBBP4	cell proliferation	Shou J (38)
	up-regulation	miR-2116-3p	SERPINA3	proliferation, invasion, inhibit apoptosis	Sun J (39)
Pancreatic cancer	up-regulation	–	EZH2, LSD1	cell proliferation	Lian Y (28)
NPC	up-regulation	miR-519	PD-L1,HIF-1α	proliferation, migration, invasion	Wang S (63)
PTC	up-regulation	miR-520c-3p	S100A4	migration, invasion	Xia F (16)
	up-regulation	miR-15a-5p	HOXA3	proliferation, migration, invasion	Jiang L (15)
OSCC	up-regulation	miR-567	CDK8	cell proliferation	Chen R (13)
	up-regulation	–	EZH2	proliferation, migration, invasion	Zhao Z (12)

NSCLC, non-small cell lung cancer; CRC, colorectal cancer; PTC, papillary thyroid cancer; NPC, nasopharyngeal carcinoma; OSCC, oral squamous cell carcinoma; HCC, hepatocellular carcinoma.

Based on bioinformatics analysis, we predicted and functionally annotated the target genes of *HOXA-AS2*. Gene ontology and KEGG pathway enrichment analysis found that target genes were mostly enriched in pri−miRNA transcription by RNA polymerase II, cell fate commitment, regulation of mRNA metabolic process, DNA−binding transcription activator activity, transcription regulator complex, transcriptional misregulation in cancer, and signaling pathways regulating pluripotency of stem cells. These functions mainly included transcriptional regulation and enzyme metabolism. Our results were in consistent with previous reports that *HOXA-AS2* was involved in tumor progression by regulating various pathways, which broadened the prospects for further study on *HOX-AS2* target therapy.

In addition to the close relationship between lncRNAs and tumor progression and metastasis, an increasing number of studies have also demonstrated that they play an important role in determining cancer cell malignant phenotypes and drug resistance (64–66). To improve the survival of patients with malignant tumors, it is imperative to understand the relationship between lncRNAs and drug resistance. As a next step, we investigated the correlation between *HOXA-AS2* expression and drug sensitivity. In acute myeloid leukemia (ALL), Zhao et al. reported that *HOXA-AS2* upregulation can reduce glucocorticoid sensitivity by regulating HOXA3/EGFR/Ras/Raf/MEK/ERK pathway (67). Dong et al. found that *HOXA-AS2* overexpression increases the resistance of acute myeloid leukemia cells to adriamycin, perhaps through the miR-520c-3p/S100A4 pathway (68). However, there has been no related research on the relation between *HOXA-AS2* and drug sensitivity or resistance in solid cancers until now. We explored the association between *HOXA-AS2* expression and drug sensitivity *via* the CellMiner™ database and found that *HOXA-AS2* expression was correlated with many drug sensitivities, such as AS-703569, pyridoclax, ON-123300, and benzaldehyde. Therefore, we deduced that *HOXA-AS2* may affect chemotherapy and may be correlated with chemoresistance. Despite of the need for further research into the mechanism of *HOXA-AS2* and drug resistance to these drugs, this finding provides us with a potential research avenue for modulating drug resistance and eventually improving cancer prognosis through inhibition of *HOXA-AS2* expression.

This large-sample study aims to investigate the correlation between abnormal expression of *HOXA-AS2* and tumor prognosis. It also revealed the characteristics of *HOXA-AS2* in multiple aspects, including expression pattern, survival prognosis, signaling pathway, and drug sensitivity. *HOXA-AS2* might serve as a potential target for cancer treatment since it displayed abnormal expression in multiple cancers and predicted a worse prognosis in cancer patients. To ensure quality and high representativeness, all relevant data should be collected as much as possible using appropriate statistical software and statistical methods. However, this study also has several limitations. 1. Among the included literature, some studies provided HRs and 95% confidence intervals directly, while others provided only survival curves, which caused a certain reporting bias. 2. Due to language limitations, the literature sources used in this study were all English literature, and related literature in other languages was not included, so the conclusion represents only a fraction of the population. 3. The included literature was a retrospective study, and the quality level of the literature may be low. 4. Many studies with positive results are more likely to be published than those with negative results, which leads to certain publication biases. 5. The included studies were all from China, so the results may only be applicable to Chinese or Asian populations. 6. We only predicted the molecular mechanisms and biological functions of *HOXA-AS2 via* GO enrichment and KEGG analysis, and the drug sensitivity analysis was carried out only by the tumor call line set using online databases. Regulatory mechanisms at a broader level should now be investigated. Overall, studies with larger sample sizes are needed to explore other potential functions of *HOXA-AS2* and its role in pro-oncogenic signaling. Using this information, cancer patients might be able to develop new therapeutic strategies.

## 6 Conclusion

High expression levels of *HOXA-AS2* are associated with poor cancer prognosis among Chinese patients, and *HOXA-AS2* may serve as an underlying therapeutic target and a promising prognostic biomarker.

## Data availability statement

The original contributions presented in the study are included in the article/**Supplementary Material**. Further inquiries can be directed to the corresponding author.

## Author contributions

YZ and QW design the project; CD and SL searched databases and performed literature screen; CD and QW extracted and analyzed the data, analysis; WZ and QW evaluated the quality of included literature; YZ, QW, WZ, and CD contributed to writing the manuscript. Final draft was approved by all the authors. All authors contributed to the article and approved the submitted version.

## Conflict of interest

The authors declare that the research was conducted in the absence of any commercial or financial relationships that could be construed as a potential conflict of interest.

## Publisher’s note

All claims expressed in this article are solely those of the authors and do not necessarily represent those of their affiliated organizations, or those of the publisher, the editors and the reviewers. Any product that may be evaluated in this article, or claim that may be made by its manufacturer, is not guaranteed or endorsed by the publisher.

## Supplementary material

Supplementary Figure 1 The publication bias of HOXA-AS2 related research.

Supplementary Table 1 Quality assessment of eligible studies Newcastle-Ottawa scale (NOS).

Supplementary Table 2 Interactional Protein-coding Genes sceened by RNAinter database.
