# Supplementary figures and images for: HOXA-AS2 may predict the prognosis of solid tumors among Chinese patients: A meta-analysis and bioinformatic analysis
Source: Front Oncol. 2022 Oct 31;12:1030825. doi: 10.3389/fonc.2022.1030825 (PMC9659612; doi:10.3389/fonc.2022.1030825)

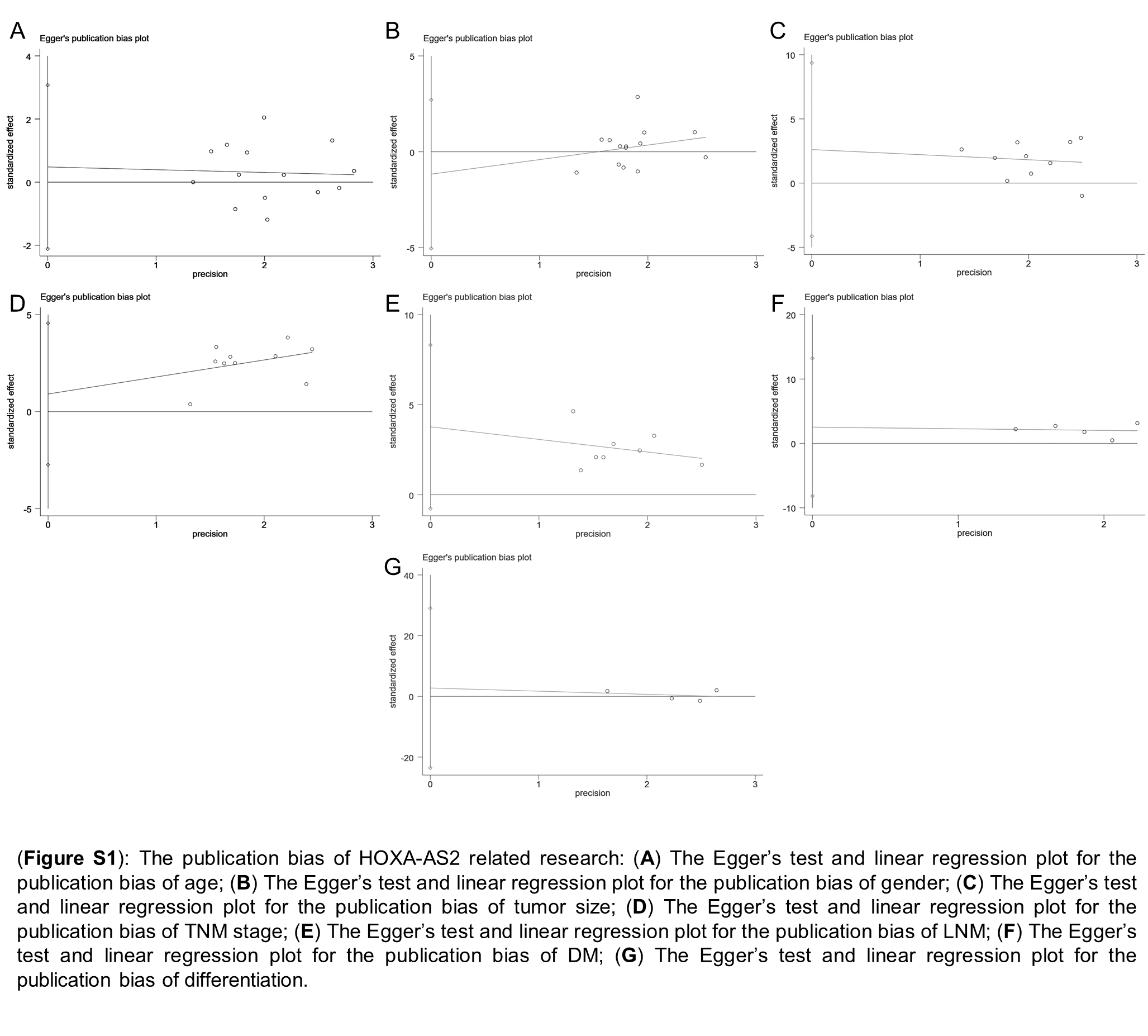

Supplement: Supplementary Figure 1 — The publication bias of HOXA-AS2 related research. [file Image_1.tif]
