# Supplementary material for: HOXA-AS2 may predict the prognosis of solid tumors among Chinese patients: A meta-analysis and bioinformatic analysis
Source: Front Oncol. 2022 Oct 31;12:1030825. doi: 10.3389/fonc.2022.1030825 (PMC9659612; doi:10.3389/fonc.2022.1030825)
Supplement: Supplementary Table 1 — Quality assessment of eligible studies Newcastle-Ottawa scale (NOS). [file Table_1.doc]

**Supplementary TableS1** Quality assessment of eligible studies Newcastle-Ottawa scale (NOS)

| Author | Country | **Selection** | | | | **Comparability** | **Outcome** | | | **Total** |
| --- | --- | --- | --- | --- | --- | --- | --- | --- | --- | --- |
|  |  | Adequate of case definition | Representativeness of the cases | Selection of Controls | Definition of Controls | Comparability of cases and controls | Ascertainment of exposure | Same method of ascertainment | Non-Response rate |  |
|  |  |  |  |  |  |  |  |  |  |  |
|  |  |  |  |  |  |  |  |  |  |  |
| Xie M (2015) | China | * | * | * | * | * | * | * | NA | 7 |
| Li Q (2016) | China | * | * | NA | * | * | * | * | * | 7 |
| Ding J (2017) | China | * | * | NA | * | * | * | * | NA | 6 |
| Wang F (2016) | China | * | * | * | * | * | * | * | NA | 7 |
| Zhang Y (2018) | China | * | * | NA | * | * | * | * | NA | 6 |
| Lu Q (2020) | China | * | * | * | * | ** | * | * | NA | 8 |
| Wang Y (2018) | China | * | * | NA | * | * | * | * | NA | 6 |
| Wang L (2019) | China | * | * | NA | * | * | * | * | * | 7 |
| Wu L(2019) | China | * | * | NA | * | * | * | * | NA | 6 |
| Fang Y (2017) | China | * | NA | * | * | * | * | * | * | 7 |
| Li Y (2017) | China | * | * | * | * | ** | * | * | * | 9 |
| Cui T J (2019) | China | * | * | * | * | * | * | * | * | 8 |
| Xia F (2018) | China | * | * | NA | * | * | * | * | NA | 6 |
| Jiang L (2019) | China | * | * | NA | * | * | * | * | * | 7 |
| Wang F (2019) | China | * | * | NA | * | * | * | * | NA | 6 |
| Xiao S (2020) | China | * | * | * | * | * | * | * | NA | 7 |
| Chen R(a) (2021) | China | * | * | NA | * | * | * | * | NA | 6 |
| Chen R(b)(2021) | China | * | * | * | * | * | * | * | * | 8 |
